# Novel technique for closure of defect in laparoscopic ventral hernia repair

**DOI:** 10.4103/0972-9941.68580

**Published:** 2010

**Authors:** Deborshi Sharma, Vikas Jindal, Om Prakash Pathania, Shaji Thomas

**Affiliations:** Department of Surgery, Lady Hardinge Medical College, New Delhi ‐ 110 001, India

**Keywords:** Defect closure, interrupted sutures, laparoscopic incisional hernia repair

## Abstract

Laparoscopic repair of ventral hernia is the standard of care in today’s era. With increasing experience, different theories and techniques have been described by different authors to overcome the intraoperative and postoperative problems. We describe a novel technique for closure of defect in laparoscopic hernia repair which has the added advantage.

## INTRODUCTION

Along with advances in minimally invasive surgery, the laparoscopic repair of incisional hernia has been gaining popularity. Yet, the absence of recurrence, seroma, and pain eludes the laparoscopic surgeon tackling ventral and incisional hernias laparoscopically[1–3] Many theories and techniques have been put forward to tackle these problems, among which is the additional step of suturing the defect.[4]

Most large published series have defined that the defect is closed by continuous suturing with a No. 1 synthetic monofilament suture or loop before the onlay mesh.[4] Authors claim stability of the repair along with the absence of a postoperative cough impulse at the site of the hernia, reduction in incidence of seroma formation, reduced size of the prosthesis required to cover the defect, and reduction of the recurrence rate.[4]

Initially in our series we had not sutured the defect before fixing the onlay mesh in order to perform a tension-free repair, which had a very high incidence of visible postoperative impulse along with seroma formation [Table 1]. Subsequently, as per the recent literature, we followed with double-layered continuous suturing, with a No. 1 loop monofilament suture. This gave us good results in terms of absence of postoperative impulse and seroma formation, however, the procedure took almost double the operating time and was associated with numerous technical difficulties and difficult suture handling experiences, such as, occasional irreversible knotting of the long suture, suture giving way during the procedure, due to inadvertent crushing with the laparoscopic instruments, necessitating re-suturing the whole defect and frequently patients complained of a foreign body sensation at the site of the final knot, which is tied in the subcutaneous plane in such a technique.

**Table 1:** Table comparing our results showing comparison between the three groups

	No suturing of defect (N = 58)	Continuous Suturing (N = 5)	Interrupted mattress suturing (N = 12)
Average size of defect (Sq cm)	15.8	16.5	16.2
Average operating time (Min)	95	165	128
Presence of cough impulse at hernia site post-operatively	12	Nil	Nil
Seroma beyond eight weeks	8	Nil	Nil
Pain persisting beyond post-operative day seven	11	1	1
Recurrence	2	Nil	Nil
Maximum Follow-up	52 months	22 months	14 months

## OUR MODIFICATION

We modified the technique of suturing to interrupted FAR-NEAR-NEAR-FAR vertical mattress sutures. It gave us the benefit of the double-layered repair, with less length of suture used, and handling became easier. Generally we used a No. 1 polypropylene monofilament suture on a 40 mm large round bodied needle, at intervals of 1/1.5cm from each other.

### Proper Technique:

**Figure 1:**
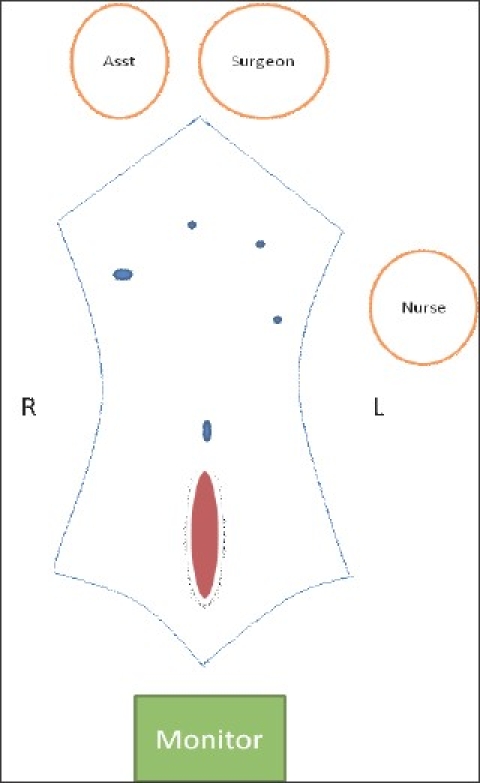
Schematic diagram showing patient, monitor and port positions for an infra-umbilical hernia

**Figure 2:**
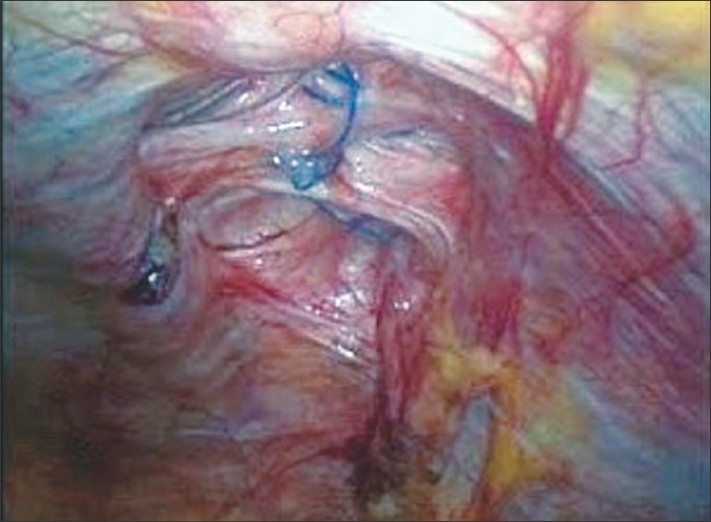
Intra-operative photograph showing the large infra-umbilical incisional hernia defect.

Patient is supine, arms tucked at the side. Monitor is at the foot end, surgeon at the head end and the patient 30 degrees head down in case of infra-umbilical hernias and vice versa in case of supra-umbilical hernias. Pneumoperitoneum is created from the palmers point and ports are inserted as shown in Figure 1. After adhesiolysis the defect size is measured for the records [Figure 2]. A 30-cm long polypropylene No. 1 suture mounted on a 40 mm needle is introduced into the peritoneal cavity by directly puncturing through the thinned out hernial sac.

**Figure 3:**
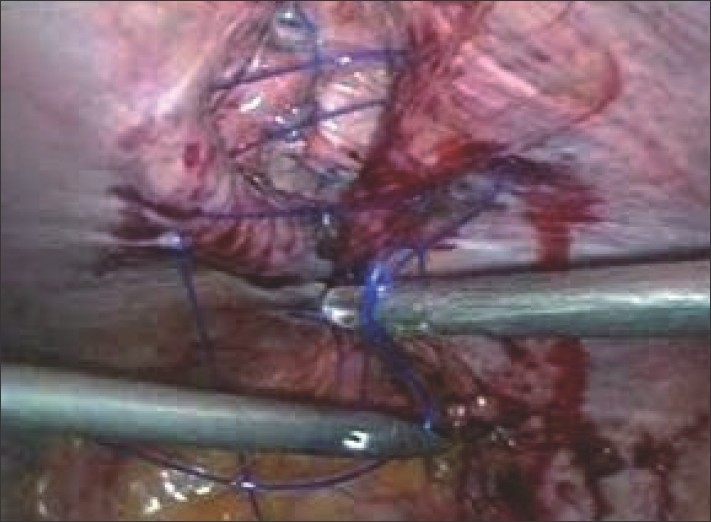
Intra-operative photograph showing suturing in progress with the interrupted far-near-near-far technique.

**Figure 4:**
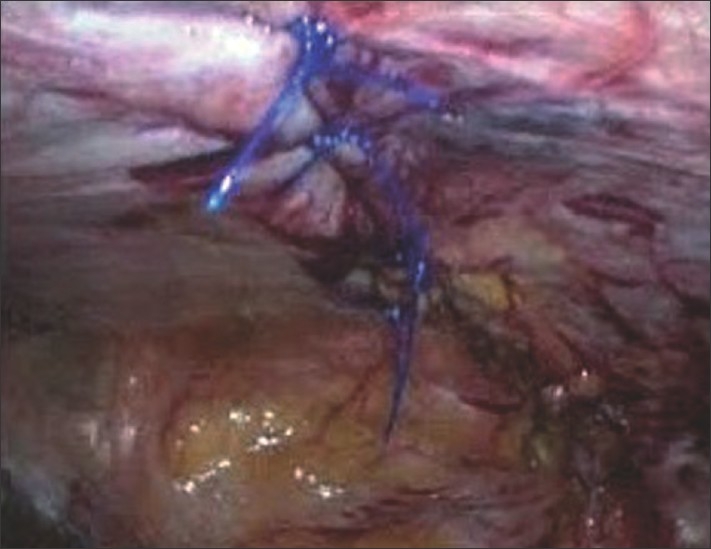
Intra-operative photograph showing the completed repair with all the polypropylene knots towards the peritoneal cavity.

**Figure 5:**
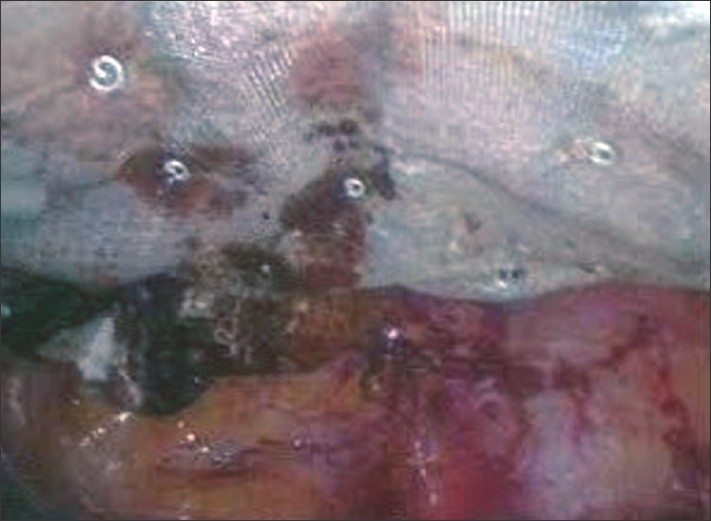
Width (Px): 295, Height (Px): 219 Color Depth: Intra-operative photograph showing the area being covered with a 15 × 15 cm tissue separating mesh.

Suturing of the edges of the defect is started at the distal end of the defect and proceeded proximally in a far-near-near-far vertical mattress suture technique [Figure 3]. Between each suture, a gap of 1 / 1.5 cm is kept. The second last suture is held with a Liga clip, to keep the end of the defect relaxed, and after completion of the last suture we tie it. The initial suture length is taken at 30 cm and if required a second suture is used. In all 12 cases so far, we could close the entire defect adequately. All knots are tied towards the peritoneal cavity preventing any subcutaneous foreign body sensation [Figure 4]. In all cases the repair is covered adequately with a multilayered tissue separating mesh [Figure 5]. The multilayered meshes we use have components of polypropylene, PDS and oxidized regenerated cellulose. The mesh before absorption gets embedded in the tissue of the anterior abdominal wall, which should further engulf the prolene knot in the abdominal wall.

## BENEFITS

This modification is beneficial in terms of reduction of operating time from that when continuous suturing is performed with the loop suture, preventing mid-suturing give way as all the sutures and knots were primarily separate with less knot pressure, better handling of smaller length sutures and prevention of subcutaneous foreign body sensation. Otherwise it has all the benefits of suturing the defect, such as, absence of postoperative impulse and significant reduction of seroma formation.
